# Contrast Enhanced Ultrasound of a Gallbladder Lesion in a Patient with a History of Renal Cell and Rectal Cancer

**DOI:** 10.1155/2013/538534

**Published:** 2013-06-27

**Authors:** Markus Reiser, Frank Oehmen, Hajo Walter, Martin Büsing

**Affiliations:** ^1^Department of Internal Medicine and Gastroenterology, Klinikum Vest GmbH, Lipper Weg 11, 45770 Marl, Germany; ^2^Department of Pathology, Mühlenstraße 31, 45659 Recklinghausen, Germany; ^3^Department of Surgery, Klinikum Vest GmbH, Dorstener Straße 151, 45657 Recklinghausen, Germany

## Abstract

The gallbladder is an uncommon site of metastatic cancer. Although ultrasound can be regarded as a first line investigation for the detection of gallbladder lesions, differentiation between benign and malignant tumors usually requires resection. Real-time contrast enhanced ultrasound (CEUS) is a well-established technique for the classification of liver, pancreatic, and renal diseases (Weskott, 2008). The application of CEUS in the diagnosis of gallbladder tumors has rarely been described. We report the application of contrast enhanced ultrasound for the characterization of a gallbladder lesion in a 63-year-old patient with a history of renal cell and rectal cancer.

## 1. Introduction

Polypoid lesions of the gallbladder are frequently found during abdominal ultrasound. The majority of these lesions represent benign cholesterol polyps and are less than 10 mm in size [1]. Primary adenocarcinoma of the gallbladder is an uncommon malignancy. Metastatic cancer to the gallbladder is also a rare finding occurring most often in melanoma, gastric cancer, and renal cell carcinoma [2]. Preoperative differentiation between benign and malignant gallbladder tumors is often difficult. This paper describes a case of a gallbladder lesion in a patient with a history of rectal and renal cell carcinoma.

## 2. Case Report

**Figure 1:**
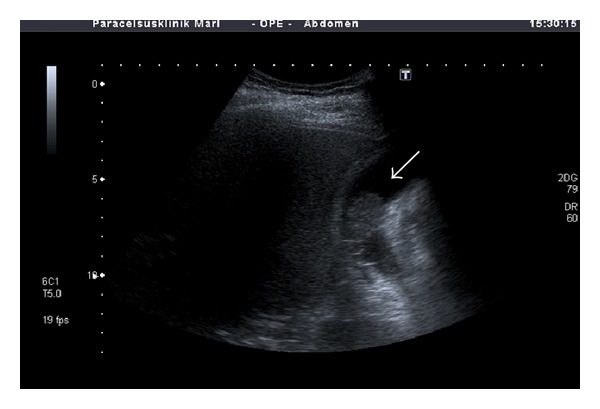
B-mode sonography showing a 25 mm polypoid lesion within the gallbladder (arrow). The tumor appears to have broad contact to the gallbladder wall at two locations.

**Figure 2:**
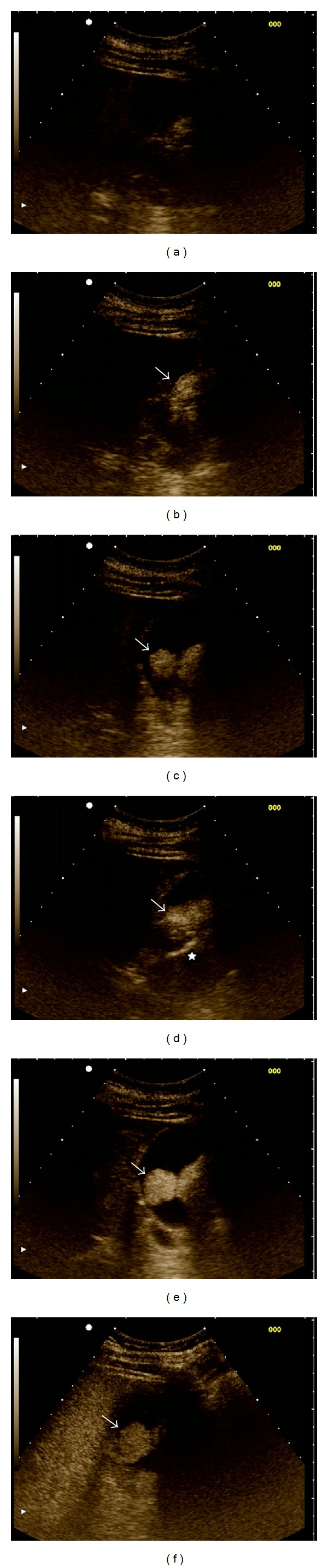
Contrast enhanced ultrasound (CEUS) of the gallbladder at various timepoints of contrast injection: (a) 0 sec (baseline); (b) 15 sec; (c) 23 sec; (d) 26 sec; (e) 35 sec; (f) 120 sec. The ultrasound monitor was set to sepia color mode for better contrast visualization. The lobulated gallbladder lesion shows intense and prolonged contrast enhancement (arrow). A feeding arterial vessel is shown in (d) (asterisk). See Supplementary Video Sequence.

**Figure 3:**
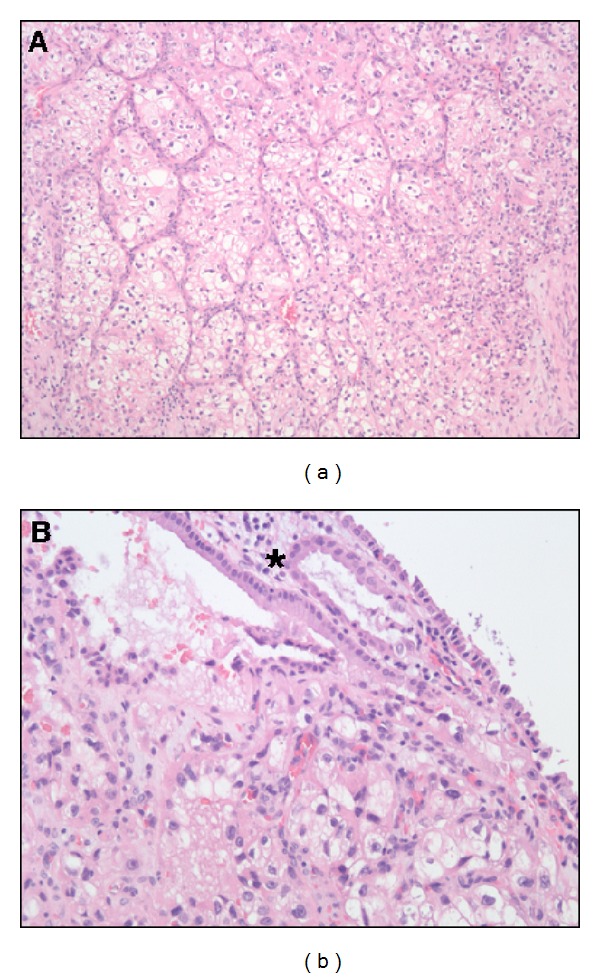
Histological examination of the gallbladder (hematoxylin eosin, (a) 100x and (b) 200x) showing metastatic clear cell renal cell carcinoma within the mucosa of the the gallbladder wall. The mucosa of the gallbladder wall is marked with an asterisk in (b).

A 63-year-old Caucasian man was admitted to our hospital for further workup of a solitary pulmonary lesion. A simultaneous diagnosis of stage I renal cell carcinoma of the left kidney and adenocarcinoma of the rectum had been made in March 2005 which was treated by left nephrectomy, total mesorectal resection, and adjuvant radiochemotherapy according to the AIO protocol. Postoperative tumor staging and grading of the rectal carcinoma was pT3, pN0 (0/15), G2, R0, and M0. Regular followup until July 2010 was without pathological findings and the carcinoembryonic antigen (CEA) and CA19-9 serum concentrations were normal. A computed-tomography-guided core biopsy of the pulmonary lesion was performed; histological and immunohistochemical analysis showed an adenocarcinoma consistent with metastatic colon cancer. On further workup, an abdominal ultrasound showed a 25 mm polypoid lesion within the gallbladder (Figure 1). Contrast enhanced ultrasound was performed for further characterization. Imaging in low mechanical index (MI) technique after intravenous injection of 5 mL sulphur hexafluoride microbubbles (SonoVue) showed an intense homogenous contrast signal within the gallbladder mass. Contrast enhancement was detected after 20 seconds and preceded the appearance of contrast in the adjacent liver tissue by approximately 20 seconds. The contrast signal decreased over time but no complete washout phenomenon was observed (see Figure 2 and Supplementary Video Sequence in Supplementary Material available online at http://dx.doi.org/10.1155/2013/538534). The diagnostic and therapeutic options were discussed by the multidisciplinary tumor board and a decision was made to resect the pulmonary metastasis and to perform a cholecystectomy. While the pulmonary tumor was confirmed to represent metastatic colon cancer, an intramucosal metastasis of a clear cell renal cell carcinoma was found in the gallbladder (Figure 3). After successful complete resection of both metastatic lesions, the patient remained without recurrent disease for more than two years. In November 2012, a computed tomography (CT) scan showed hepatic metastases which were considered metastatic renal cell carcinoma because of the radiologic appearance. The patient was therefore treated with pazopanib. Followup in February 2013 showed marked tumor regression.

## 3. Ultrasound Technique

Contrast enhanced ultrasound (CEUS) was performed after baseline examination using an EUB 6500, Hitachi Medical System. The imaging mode was changed to CEUS with a low mechanical index of <0.1. Five ml sulphur hexafluoride microbubbles was administered via the antecubital vein in a bolus fashion (within 1-2 s), followed by a flush of 5 mL of 0.9% normal saline by using a 20-gauge cannula. Imaging was recorded continuously for a period of 120 seconds.

## 4. Discussion

Metastatic cancer of the gallbladder is a rare finding only occasionally seen in malignant melanoma, ovary, and gastrointestinal malignancies. Metastatic renal cell carcinoma of the gallbladder has also been described in some case reports [3–5]. The typical diagnostic sequence begins with the detection of a polypoid lesion by routine ultrasound. Followup is usually recommended for polyps less than 10 mm; however, further workup is needed for larger tumors especially in patients with a history of malignant diseases. CT or magnetic resonance imaging may help to characterize gall bladder lesions; still, differentiating between benign (e.g., cholesterol polyps or sludge) and malignant tumor (primary cancer or metastases) is difficult and surgical resection is usually needed for making a definite diagnosis.

Real-time CEUS is best established for the characterization of liver lesions but has been applied for a growing number of indications such as renal, prostate, breast, and splenic diseases [6, 7]. CEUS has also been shown to be helpful in differentiating malignant gallbladder lesions from benign alterations such as biliary sludge or chronic cholecystitis with thickened gallbladder wall. Most reports describe CEUS in primary gallbladder carcinomas showing early hyper- or isoenhancement of the tumors followed by washout within 35 seconds after contrast agent administration [8, 9]. Only few case reports have been published on CEUS in metastatic gallbladder disease, most of which were metastatic melanoma. A moderate to intense contrast enhancement in the arterial phase and a more or less rapid washout in the venous phase have been described in these cases; a branching pedicle was sometimes recognizable [10]. In the present case of metastatic renal cell carcinoma, CEUS showed comparable findings with intense contrast enhancement in the arterial phase but without significant washout in the venous phase. In addition, a feeding arterial vessel could be identified. Although these findings clearly indicate the presence of a well-vascularized tumor, benign lesions such as adenomas cholesterol and inflammatory polyps are supplied by arterial branches of the cystic artery and may also show contrast enhancement in the early phase of CEUS.

In conclusion, metastatic cancer involving the gallbladder is a rare condition which may be seen in malignant melanoma, gastrointestinal cancer, and renal cell carcinoma as presented in this case report. CEUS is a valuable technique for characterizing gallbladder lesions. However, CEUS has to be interpreted in the clinical context. Surgery may be needed to make a definite diagnosis.

## Supplementary Material

Description for video sequence:
Contrast enhanced ultrasound (CEUS) of the gallbladder (12-38 sec after injection). A low mechanical index of < 0,1 was used and the ultrasound monitor was set to sepia colormode for better contrast visualization. Five ml sulphur hexafluoride microbubbles (Sonovue®) were administered via the antecubital vein. Intense and prolonged contrast enhancement of the gallbladder lesion is best seen from 24 sec on (please note time in right upper corner).
